# Distinct Control of Initiation and Metrics of Memory-Guided Saccades and Vergence by the FEF: A TMS Study

**DOI:** 10.1371/journal.pone.0020322

**Published:** 2011-05-26

**Authors:** Qing Yang, Zoi Kapoula

**Affiliations:** IRIS Group, UMR 8194, CNRS, Hôpital Européen Georges Pompidou, Paris, France; Université Pierre et Marie Curie, France

## Abstract

**Background:**

The initiation of memory guided saccades is known to be controlled by the frontal eye field (FEF). Recent physiological studies showed the existence of an area close to FEF that controls also vergence initiation and execution. This study is to explore the effect of transcranial magnetic simulation (TMS) over FEF on the control of memory-guided saccade-vergence eye movements.

**Methodology/Principal Findings:**

Subjects had to make an eye movement in dark towards a target flashed 1 sec earlier (memory delay); the location of the target relative to fixation point was such as to require either a vergence along the median plane, or a saccade, or a saccade with vergence; trials were interleaved. Single pulse TMS was applied on the left or right FEF; it was delivered at 100 ms after the end of memory delay, i.e. extinction of fixation LED that was the “go” signal. Twelve healthy subjects participated in the study. TMS of left or right FEF prolonged the latency of all types of eye movements; the increase varied from 21 to 56 ms and was particularly strong for the divergence movements. This indicates that FEF is involved in the initiation of all types of memory guided movement in the 3D space. TMS of the FEF also altered the accuracy but only for leftward saccades combined with either convergence or divergence; intrasaccadic vergence also increased after TMS of the FEF.

**Conclusions/Significance:**

The results suggest anisotropy in the quality of space memory and are discussed in the context of other known perceptual motor anisotropies.

## Introduction

Short-term memory, also called working memory, is a high level cognitive process of limited capacity [1]. It has generated much interest recently because of its importance to many higher brain functions and the evolution of powerful techniques to study brain function, such as event-related potentials [2], positron emission tomography [3], functional Magnetic Resonance Imaging [4] and transcranial magnetic stimulation [5]. Memory-guided saccade paradigm has been used extensively since then for studies in animals [6], in normal humans [7] and in pathology [8]. Pierrot-Deseilligny et al. [9], [10] proposed the following hypothetical circuitry: After passing several occipital visual areas visual information is integrated in spatial coordinates within the posterior parietal cortex (PPC); the result of this integration is probably sent, through cortico-cortical connections to prefrontal cortex, where it is stored using short-term memory. The next relay is frontal eye field (FEF), which finally descends the information to superior colliculus and/or to paramedian pontine reticular information to trigger the memory-guided saccades. [9], [10]

The large majority of studies concern memory-guided saccades [9], [11], [12] and to our knowledge, not many on memory-guided movements in depth, i.e. vergence [13]. The latter is the movement allowing to adjust the angle of visual axes according to distance of the object. Under normal visual conditions, vergence eye movements are stimulated by several cues such as accommodation, proximity and most important binocular depth cues such as disparity. Subjects can also produce any ocular vergence responses by attempting binocular fixation of an imaged target moving back and forth in darkness [14]. Here we introduce a paradigm with targets flashed at unpredictable locations calling either for a saccade, or a vergence, or combined saccade-vergence movements. Such stimuli interleaving depth and direction components reproduce better natural situations and needs short term memory of targets.

The FEF controls not only saccades but also vergence eye movements. Jampel [15] reported that stimulation of the frontal lobe could elicit both saccade and vergence eye movements. Recently, Gamlin et al. (1996, 2000) characterized neurons in a prearcuate area related not only to either the far response or the near response, but also to the sensorimotor transformation underlying these eye movements. Moreover, Ferraina et al. [16] found that 2/3 of FEF visual and visuo-movement neurons were sensitive to disparity and showed a broad tuning in depth for near or far disparities. More recently, Kurkin et al. [17] reported that caudal parts of the FEF contained smooth pursuit neurons and the discharge of the majority of them was related to vergence eye movements as well. The purpose of this study is to explore the role of FEF in the control of such memory-guided saccade, vergence and combined movements; also to explore the possible TMS effect on intrasaccadic vergence called saccade disconjugacy.

## Results

### Effect of TMS on the latency of eye movements

**Figure 1:**
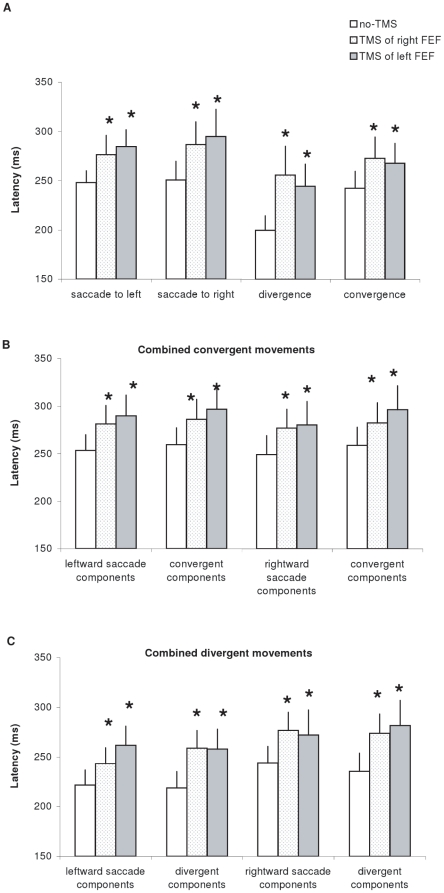
Mean values of latency with standard error. (A) for saccades to left, saccades to right, divergence and convergence, (B) for combined convergent movements and (C) for combined divergent movements (C) under the three experimental conditions: no-TMS, TMS over the right FEF and TMS over the left FEF. Asterisks indicate significant increases of latency after TMS over left or right FEF relative to no-TMS (p<0.05).

**Figure 2:**
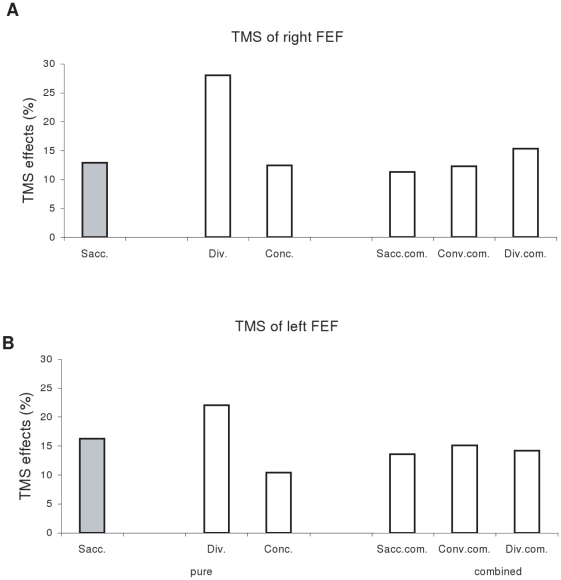
Mean values of percentage of TMS effects in latency, (TMS-noTMS)/noTMS. (A) TMS of right FEF and (B) TMS of left FEF for divergence, saccades, convergence, and saccade components, convergence components and divergence components of combined movements under the conditions of TMS over the right or the left FEF. Such value is higher for divergence than for any other types of eye movements.

Figure 1 presents the group mean latency for saccades to left or to right, for divergence and convergence (A), for components of combined convergent movements (B) and for components of combined divergent movements (C); data are shown under no-TMS, TMS over the right or left FEF conditions. Two-way ANOVA showed significant main effect of the TMS (F_(11,121)_ = 6.66, p<0.001), i.e., longer latency after TMS over the right or the left FEF relative to no-TMS; and also significant effect of the type of eye movement (F_(2,22)_ = 21.69, p<0.001). Post-hoc analysis showed that the effect of TMS was significant for each type of eye movement: saccade, vergence or combined eye movements (all p<0.05). Divergence showed significantly shorter latency than any other type of eye movements (all p<0.05). We calculated the percentage of latency changes after TMS, i.e. (TMS - noTMS)/noTMS, and found that such percentage was higher for divergence (28% for TMS of right FEF, 23% for TMS of left FEF) than other type of eye movements (from 12% to 16%, see Figure 2). This suggests that TMS effect is stronger for the movements which have naturally the shortest latency, such as divergence.

### Effect of TMS on the percentage of error in amplitude of eye movements

**Figure 3:**
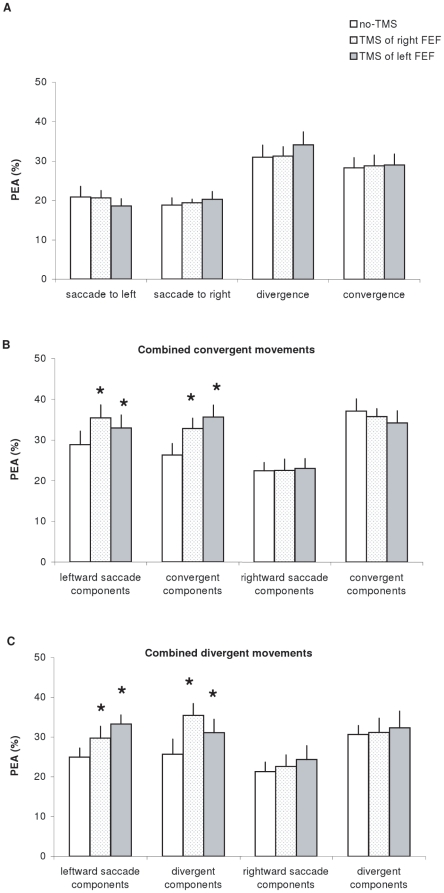
Mean values of percentage error amplitude (PEA) with standard error. (A) for saccades to left, saccades to right, divergence and convergence, (B) for combined convergent movements and (C) for combined divergent movements under the three experimental conditions: no-TMS, TMS over the right, or the left FEF. Asterisks indicate significant increases of PEA after TMS over the left or the right FEF relative to no-TMS.

Figure 3 presents the group mean PEA for saccades to left or to right, for divergence and convergence (A), for components of combined convergent movements (B) and for components of combined divergent movements (C) under three conditions: no-TMS, TMS over the right or the left FEF. The **Friedman** test applied on the PEA under no-TMS condition showed significant effect of type of eye movement (Chi^2^_2,12_ = 37.2, p<0.001). For pure eye movements, vergence (both divergence and convergence) showed higher PEA than pure saccades (both leftward and rightward, all p<0.05, Wilcoxon test). For combined eye movements, only leftward saccade components of combined convergent movements and convergent components of combined leftward movements showed higher PEA than their corresponding pure eye movements (both p<0.05).

The Friedman test applied on TMS condition separately for each type of eye movement showed significant TMS effect for combined saccades to left with convergent movements (for both their saccade components Chi^2^_2,12_ = 9.5, p<0.01 and their convergence components p<0.01 and Chi^2^_2,12_ = 12.67, p<0.01); also for combined saccades to left with divergent movements (for both their saccade components Chi^2^_2,12_ = 8.17, p<0.05 and their convergence components Chi^2^_2,12_ = 11.17, p<0.01). The Wilcoxon test used for two by two comparisons showed that for all such combined movements to the left the PEA relative to no-TMS was significantly higher after either TMS of the left FEF or after TMS of the right FEF (p<0.05).

**Figure 4:**
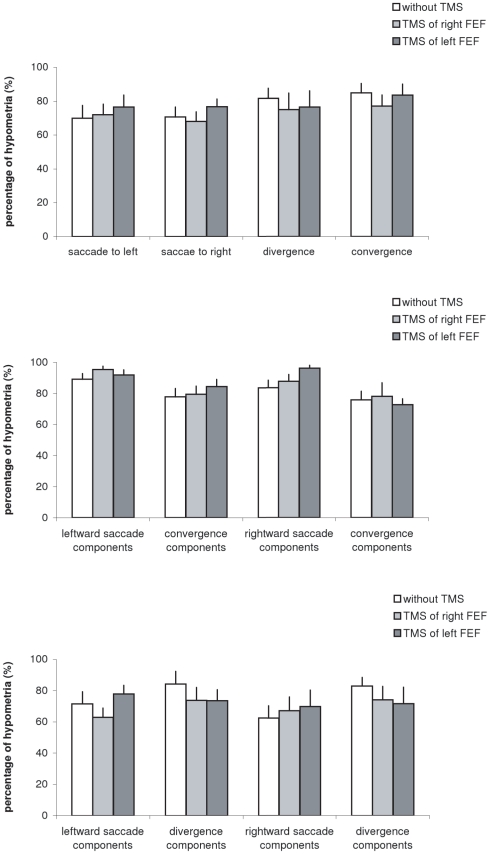
Mean values of percentage of hypometria with standard error. (A) for saccades to left, saccades to right, divergence and convergence, (B) for combined convergent movements and (C) for combined divergent movements under the three experimental conditions: no-TMS, TMS over the right, or the left FEF.

Note that the majority of eye movements are hypometric. Figure 4 presents the group mean percentage of hypometria for each type of eye movement under different TMS conditions. Non-parametric statistical analysis showed no significant difference in the percentage of hypometria between any two types of eye movements, or between any no-TMS and TMS conditions (all p>0.05).

### Effect of TMS on the intrasaccadic vergence changes

**Figure 5:**
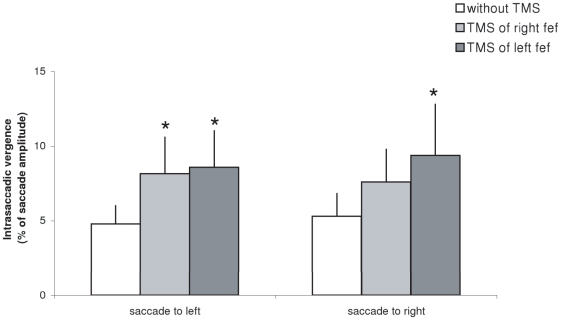
Mean values of intrasaccadic vergence with standard error. Asterisks indicate significant increases of intrasaccadic vergence after TMS of the left or the right FEF relative to no-TMS.

Figure 5 presents the group mean intrasaccadic vergence (expressed as a percentage of saccade amplitude) for saccades to left or to right under three conditions: no-TMS, TMS over the right or the left FEF. The Friedman test showed significant TMS effect for saccades to left (Chi^2^_2,12_ = 8.2, p<0.05) and for saccades to right (Chi^2^_2,12_ = 6.5, p<0.05). For saccades to left, TMS of the left or of the right FEF caused significant increase of intrasaccadic vergence (p<0.05); for saccades to right, TMS of the left FEF increased intrasaccadic vergence significantly (p<0.05) while TMS of the right FEF increased such vergence but not significantly (p = 0.09).

### No effect on mean velocity of eye movements

**Figure 6:**
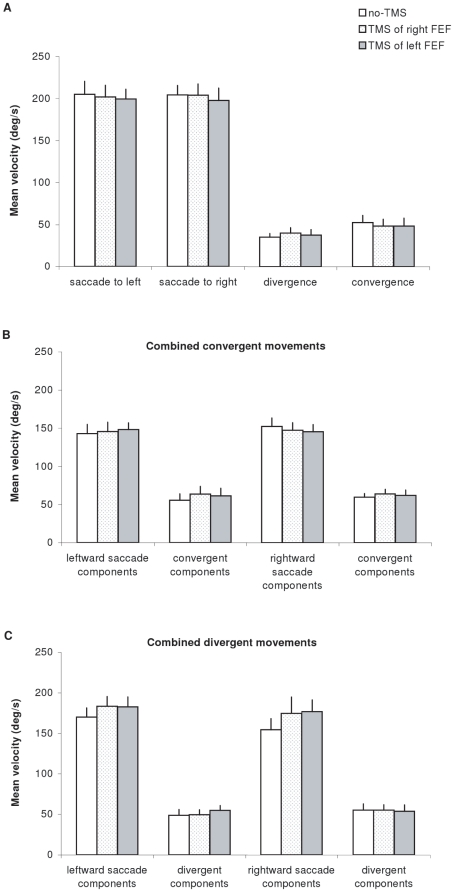
Mean values of mean velocity with standard error. (A) for saccades to left, saccades to right, divergence and convergence, (B) for combined convergent movements and (C) for combined divergent movements under the three experimental conditions: no-TMS, TMS over the right FEF and TMS over the left FEF.

Figure 6 presents the group mean values of mean velocity for saccades to the left or to the right, for divergence and convergence (A), for components of combined convergent movements (B) and for components of combined divergent movements (C) under no-TMS, TMS over the right and TMS over the left FEF conditions. One-way ANOVA test applied separately on each type of eye movement showed no significant effect of condition (all p>0.05).

## Discussion

### Increase of latency after TMS of FEF

TMS over the FEF delivering at 100 ms after the extinction of the fixation point could interfere with the fixation disengagement process. Such mechanism could explain the increase of latency of bilaterally memory-guided saccades. This interpretation is compatible with the study on patients with the FEF lesions showing also a bilateral latency increase of memory-guided saccades [18]. Another possible mechanism could be interference with activity of movement related neurons of the FEF, which is compatible with physiological studies in monkeys [12]. The important novel aspect brought by the present study is that the FEF controls the initiation of all types of memory-guided movements in the 3D space, saccades, vergence and combined movements.

Neurons of FEF has been reported involved in vergence eye movements [15], [17], [19]. One should recall, however, that here we deal with memory-guided vergence. Areas FEF could be involved in the processing the disparity of a target presented in depth, and such information must be stored to create subsequently the command signal for a voluntary memory-guided vergence. Some evidence for memory depth activity also exists from animal studies [20] has shown the existence of both visual and memory depth information. We suggest that similarly to the saccade circuitry, the FEF may play a role in providing the ‘go’ signal for triggering vergence movements in depth. Thus, we attribute the delay of latency of memory-guided vergence to TMS interference with the disengagement of fixation and perhaps with the premotor memory activity of vergence movement related neurons.

The initiation of combined eye movements could involve more complex operations as the brain should control two commands, in direction and in depth, that at least at the brainstem level, are executed by distinct, but interactive generators [21], [22]. It is interesting that TMS over the FEF produced similar effects for the two components. These observations are compatible with our previous work on the role of PPC on the initiation of combined eye movements [23]. Taken together the present and past TMS studies on saccades and vergence movements, the results indicate globally that the same structures control the initiation of saccades and vergence eye movements whatever their nature is, visually guided or memory guided.

### Selective increase of the percentage of error in amplitude after TMS of the FEF

The percentage of error in amplitude (PEA) of the primary saccades in a memory-guided task in the present study was about 19%, that is similar to that reported by other studies [24], [25]. Moreover, we found that vergence had higher PEA than saccades. This result is compatible with another study [26]. These authors used a different paradigm, the remembered-target double-step paradigm and reported that the gain of vergence of the first movement was only 60%, lower than that of the saccades, that was roughly correct, i.e; 100%.

A novel result is that TMS of the FEF did not impair the accuracy of memory-guided vergence or saccades. Nevertheless, patients with FEF lesions [18], [27] show higher PEA than healthy subjects. Thus, there is a controversy between TMS studies and studies of patients with FEF lesions as far as saccades are concerned. Perhaps this is because single pulse TMS interferes with the function of FEF only transiently, unlike lasting FEF lesions. Accuracy of saccades or vergence would thus be spared by single pulse TMS.

However, TMS over the FEF degraded the accuracy of memory-guided combined eye movements, especially for leftward saccades combined with divergence or convergence. This highly specific effect is of interest. Animal studies show movement amplitude related activity in the FEF for both saccades and vergence eye movements [19], [28]. The effect on the PEA could reflect a deterioration of such signals due to TMS interference. The specificity of this effect may be related to anisotropy of space memory. Memorizing a target location with both direction and depth components in the left visual field could be more demanding, involving more the FEF. Prior studies dealing with visually guided saccades did not show such left/right asymmetry [23]. Yet, in all these studies only the amplitude of the movement was evaluated, and not the percentage of error. As shown in Fig. 4, for the data without TMS, the PEA is least for saccades alone (left or right); when combined with vergence, the error of the saccade component increases only for the leftward saccades. Thus, even without TMS, the combination of a leftward saccade and of a combined vergence command seems to be less accurate. TMS of the FEF deteriorated further the accuracy of such movements. Perhaps this anisotropy for error amplitude is due to attention, memory, motor aspects, or combination of all. An analogy can be made with performances in other tasks such as bisection task. Left/right asymmetry has been reported in healthy subjects [29]. Moreover, Weiss et al. [30] showed that the cerebral activation is different according to the depth at which the bisection task is performed (close *vs* far).

### Increase of intrasaccadic vergence after TMS of the FEF

Under no-TMS condition, the intrasaccadic vergence of memory-guided saccades was about 5% of saccade amplitude. This is compatible with results for visually-guided saccades of similar amplitude [31]. This result is novel. Even in dark, without a visible target, the eyes make conjugate saccades keeping disconjugacy as small as that for saccades to visible targets. Another important result of this study is that TMS of the FEF can increase the intrasaccadic vergence even though no changes occur in the conjugate saccade amplitude. The exact origin of intrasaccadic vergence is not known. For visually-guided saccades peripheral origin has been suggested, such as mild asymmetry between the lateral rectus muscle of one eye and the medial rectus muscle of the other eye [21], [32]. However, this explanation is not sufficient as during the development in children, peak velocity of saccades (reflecting muscular properties) does not change, while intrasaccadic vergence decreases with age [33]. Central mechanisms are also involved in the control of intrasaccadic vergence. For instance, Vernet et al. [34] showed that TMS of the PPC increases intrasaccadic vergence for visually-guided saccades. The present result indicates that FEF is also involved in the control of intrasaccadic vergence for memory-guided saccades. We hypothesize that the central origin for intrasaccadic vergence is based on saccade vergence interaction similar to what occurs when looking between targets that are in different direction and depth. Busettini and Mays [35] provided new physiological data of such combined saccade-vergence movements and a new model according to which the acceleration of the vergence by the saccade would result from a multiplicative interaction between the position command driving the saccade system and an estimation of the vergence motor error driving the vergence system. An internal mechanism of feedback would control the movement progression and this feedback is suggested to be a cortico-midbrain-cortical loop. We suggest that the same central mechanism of continuous saccade vergence interaction applies for memory-guided saccades.

Namely with every saccade command the central nervous system programs a small but rapid vergence command aiming to reduce peripheral asymmetries of extraocular muscles. This hypothesis presented by Vernet et al. (2008) for visually-guided saccades can be expanded for memory-guided saccades. Finally, it is important to note that after TMS of the FEF the percentage of error in amplitude increased significantly only for combined saccade-vergence movements but not for pure saccades or pure vergence. Perhaps the FEF is primarily concerned with the metrics of complex movements such as combined saccade-vergence gaze shifts and also with keeping vergence appropriate during saccades.

In conclusion, the present study shows that TMS over the right or over the left FEF interferes with triggering of all movements, saccades, vergence and combined saccade-vergence driven by memory. Their latency increases after TMS of FEF. Such latency increase is attributed to TMS interference with the fixation disengagement process and/or with the premotor memory activity of saccade and vergence movement neurons of the FEF. These results suggest that both the right and the left FEF are involved in the initiation of memory-guided eye movements in 3D space. The amplitude error of such movements unaffected by TMS except for combined leftward –convergent or –divergent memory guided movements. The error increase for such movements suggests the existence of an anisotropy in the quality of space memory and/or of its motor and attention components. Increases of intrasaccadic vergence after TMS of the FEF supports the ideas, central origin of such vergence and of continues interaction of saccade vergence physical mechanism.

## Materials and Methods

### Esthetic statement

The eye movement investigation adhered to the tenets of the Declaration of Helsinki and was approved by the local human experimentation committee, CPP Il de France II (No: 07035), Hospital Necker in Paris. Written consent was obtained from all subjects after the nature of the examination had been explained.

### Subjects

Twelve healthy adult subjects, 5 females and 7 males, all right-handed, participated in the experiment (with or without TMS). Their ages ranged from 23 to 47 years (mean 34±8 years). All subjects had normal or corrected-to-normal vision. Binocular vision was assessed with the TNO test of stereoacuity; all individual scores were normal, 60″ of arc or better. Each subject gave informed consent to participate in the study.

### Visual display

The visual display consisted of LEDs placed at two isovergence circles: one at 20 cm from the subject, and the other at 150 cm. On the close circle three LEDs were used; one at the center and the others at ±20°. The required mean vergence angle for fixating any of these three LEDs was 17°. On the far circle, five LEDs were placed: one at the center, two at ±10° and two at ±20°; fixation to any of these LEDs required vergence angle of 2.3°.

### Oculomotor procedure

In a dark room, the subject was seated in an adapted chair. The subject viewed binocularly and faced the table of the LEDs which were highly visible at all target locations.

### Oculomotor tasks

#### Memory –guided eye movements

**Figure 7:**
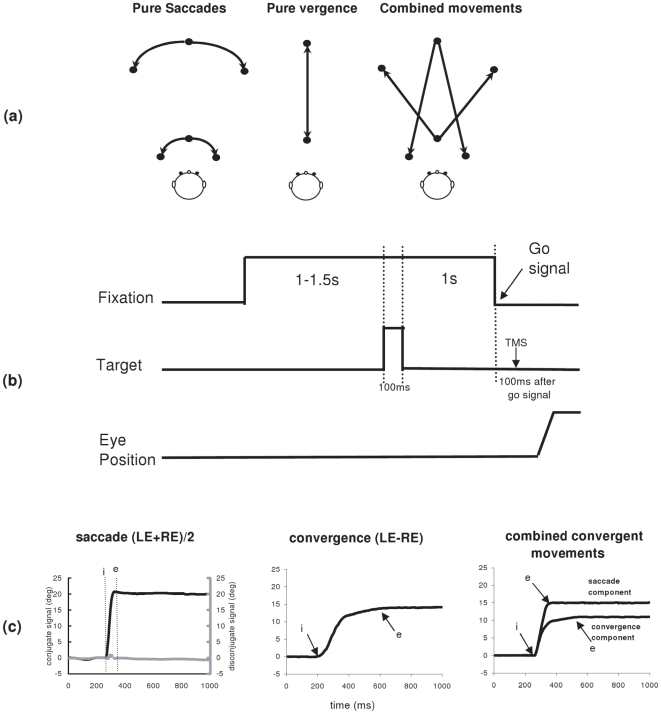
Experimental paradigms. (a) Different types of eye movements elicited; saccades, convergence and divergence along the median plane, and combined convergent or divergent movements. (b) Events during a trial of the paradigm for memory-guided eye movements. (c) Typical recordings of saccades (conjugate signal in black and disconjugate signal in grey), convergence and combined convergent movements; the conjugate signal (saccade or saccade component) is obtained by averaging the position signal of the two eyes (LE+RE)/2; the disconjugate signal (convergence, convergence component) is the difference between the two signals LE-RE. The arrows at ‘*i*’ and at ‘*e*’ indicate the onset and the end of movements, respectively.

Each trial started by lighting a fixation LED at the center of one of the circles (far or close). After a random period of 1000–1500 ms the target LED was flashed for 100 ms; the central LED remained on for 1000 ms, during which the subject was asked to remember the position of the target flashed (See Fig. 7b). After the memory delay of 1000 ms the fixation LED was turned off, and this was the ‘go’ signal for making a movement in the dark towards the remembered target location. When the flashed target-LED was on the center of the other circle called for a pure vergence eye movement, along the median plane. When it was at the same circle called for a pure saccade, and when it was lateral and on the other circle the required eye movement was a combined saccade with vergence (see Fig. 7a).

There were 10 types of memory-guided movements randomly interleaved: saccades (left or right at far or close); convergence or divergence (along the median plane); combined convergent or divergent movements to the left or to the right. All lateral target LEDs were at 20°; all targets along the median plane required a change in ocular vergence of 15°; similarly, combined movements required a saccade of 20° and a vergence of 15°.

Each subject performed 24 blocks of 40 trials, i.e. 8 blocks for each of the following conditions, TMS over the right FEF, TMS over the left FEF, no-TMS stimulation. Four sessions were performed separated by a week, and for each session 6 blocks (2 blocks for each condition) were run lasting approximately one hour. The order of the conditions was counterbalanced to neutralize fatigue effects. In total, there are 32 trials for each type of eye movement under each condition.

### Calibration task

At the beginning and end of each of the above blocks the subject made a sequence of saccades used for calibration. A LED target was presented successively at the center, left, center, right at 10° or 20°, at far (150 cm), and at near (20 cm). For each location, the LED target remained on for 2 s (a period sufficiently long to allow accurate and stable fixation); the subject was instructed to fixate the LED as accurately as possible. From these recordings were extracted the calibration factors.

### Eye movement recording

Horizontal movements from both eyes were recorded simultaneously were recorded with the EyeLink II device. Each channel was sampled at 250 Hz. The system has a spatial resolution of 0.025° in pupil-CR mode and saccade event resolution of 0.05° for microsaccades.

### TMS localization

Single-pulse TMS was applied by a MagStim 200 magnetic stimulator with a figure-of-eight coil (each wing 70 mm diameter). In separate blocks the right or the left FEF was stimulated. The localization of the FEF was done as follows. At first, the hand motor area was localized by inducing a slight muscle twitching of the contralateral hand after TMS stimulation; the stimulation threshold was thus determined. Then the coil stimulator was moved anterior the hand motor area by 2–3 cm until no muscle switching of the hand was visible. The handle of the coil was pointed backwards and the inducing current was from posterior to anterior. This method of localization of the FEF has been used by several other studies [24], [36], [37], [38].

The FEF was stimulated at 40%–55% of total stimulator output depending on the subjects, which is above motor threshold of subject's stimulation; TMS did not cause blinks (monitored on real time). The rising time of the TMS pulse was 5 µs, the decay lasting 160 µs, and a click occurred simultaneously with the stimulation discharge. TMS occurred 100 ms after the ‘go’ signal, i.e. the extinction of the central fixation LED. For reference experiments without TMS, the stimulator was switched on but the coil was placed 30 cm over the head of the subject and oriented towards the ceiling; this produced the same acoustic events as when doing effectively TMS trials. Another coil unlinked to the magnetic stimulator was placed over the subject head, in order to conserve the same somato-sensory clues as during the real stimulation.

### Data analysis

Calibration factors for each eye were extracted from the saccades recorded in the calibration task; a linear function was used to fit the calibration data. From the two individual calibrated eye position signals we derived the conjugate signal which was the mean of both eyes (left+right/2), and the disconjugate signal, i.e. the difference between two eyes (left – right); the conjugate signal is the saccade or saccade component, the disconjugate signal is the vergence or vergence component. The onset and the offset of a pure saccade or of the saccadic component of the combined movements were defined as the time when eye velocity exceeded or dropped below 10% of saccadic peak velocity. The onset and the offset of the vergence signals (for pure vergence movement or for the vergence component of the combined movements) were defined as the time point when the eye velocity exceeded or dropped below 5°/s. These criteria are standard [39], [40], [41]. The placement of the markers by the computer was verified by one of the investigators scrutinizing saccade and vergence components on the screen. From these markers, we measured the latency of eye movements, e.g. the difference between target onset and eye movement initiation. The eye movement amplitude is the position difference between the marker of end of movement (‘e’) and the marker of start of the movement (‘i’, see Fig. 1c). To estimate accuracy we measured the absolute value of the percentage of error in the amplitude of eye movements as follows: PEA = (target amplitude-memory eye movement amplitude)/target amplitude*100. The mean velocity (degree/sec) is the ratio of eye movement amplitude/duration (time difference between ‘i’ and ‘e’). Intrasaccadic vergence, the so called saccade disconjugacy was also measured; markers ‘i’ and ‘e’ were projected on the disconjugate signal (see Fig. 7C for saccade). The amplitude ‘i’ to ‘e’ of the disconjugate signal is the intrasaccadic vergence, expressed as a percentage of the conjugate saccade amplitude. Eye movements in the wrong direction, anticipatory movements (with latency shorter than 80 ms), and slow movements (with latencies longer than 1000 ms), or movements contaminated by blinks were rejected. About fifteen percent of memory-guided trials had to be rejected (individual rates 11%–20%).

Two-way ANOVA was applied on individual means for statistical analysis of latency or mean velocity of all types of eye movements between TMS and no-TMS conditions. The LSD post-hoc test was used for paired comparisons between any two conditions. Friedman and Wilcoxon tests were used for comparisons of PEA for each type of eye movement and of intrasaccadic vergence.
